# Regulator of G protein signaling 2 inhibits Gα_q_-dependent uveal melanoma cell growth

**DOI:** 10.1016/j.jbc.2022.101955

**Published:** 2022-04-19

**Authors:** Qian Zhang, Andrew J. Haak, Benita Sjögren

**Affiliations:** 1 Department of Medicinal Chemistry and Molecular Pharmacology, Purdue University, West Lafayette, Indiana, USA; 2 Department of Physiology and Biomedical Engineering, Mayo Clinic, Rochester, Minnesota, USA

**Keywords:** uveal melanoma, G protein, GTPase activating protein (GAP), regulator of G protein signaling (RGS), RGS2, cell proliferation, effector antagonism, extracellular-signal-regulated kinase (ERK), Dox, Doxycycline, ERK, extracellular-signal-regulated kinase, GAP, GTPase-activating protein, GPCR, G protein-coupled receptor, MAPK, Mitogen-activated protein kinase, RGS, Regulator of G protein signaling, Tram, Trametinib, YAP, Yes-associated protein

## Abstract

Activating mutations in Gα_q/11_ are a major driver of uveal melanoma (UM), the most common intraocular cancer in adults. While progress has recently been made in targeting Gα_q/11_ for UM therapy, the crucial role for these proteins in normal physiology and their high structural similarity with many other important GTPase proteins renders this approach challenging. The aim of the current study was to validate whether a key regulator of Gq signaling, regulator of G protein signaling 2 (RGS2), can inhibit Gα_q_-mediated UM cell growth. We used two UM cell lines, 92.1 and Mel-202, which both contain the most common activating mutation Gα_q_^Q209L^ and developed stable cell lines with doxycycline-inducible RGS2 protein expression. Using cell viability assays, we showed that RGS2 could inhibit cell growth in both of these UM cell lines. We also found that this effect was independent of the canonical GTPase-activating protein activity of RGS2 but was dependent on the association between RGS2 and Gα_q_. Furthermore, RGS2 induction resulted in only partial reduction in cell growth as compared to siRNA-mediated Gαq knockdown, perhaps because RGS2 was only able to reduce mitogen-activated protein kinase signaling downstream of phospholipase Cβ, while leaving activation of the Hippo signaling mediators yes-associated protein 1/TAZ, the other major pathway downstream of Gα_q_, unaffected. Taken together, our data indicate that RGS2 can inhibit UM cancer cell growth by associating with Gα_q_^Q209L^ as a partial effector antagonist.

Uveal melanoma (UM) is the most common primary intraocular cancer in adults, arising from melanocytes residing within the three parts of the uveal (iris, ciliary body, and choroid) (1). The disease is associated with a high mortality rate, with up to 50% of patients developing metastases with a 1-year survival rate of 15% (2). There are currently no targeted therapies for UM, and patients are limited to relying on surgery and radiation therapy, which cause severe side effects (1). Hence, in recent years, several groups have dedicated significant effort to identifying targeted UM therapeutic strategies.

A majority of UM patients (85%–91%) have a mutation in either the *GNAQ* or *GNA11* gene in a mutually exclusive pattern (3, 4). These genes encode two members of the Gq subfamily of heterotrimeric G protein α subunits, Gα_q_ and Gα_11_, respectively. These proteins share 90% protein sequence similarity and have been suggested to play similar physiological functions (5). Mutations associated with UM all occur at either glutamine 209 (Q209) or arginine 183 (R183) (3) and render Gα_q/11_ constitutively active. This, in turn, results in constitutive activation of multiple downstream effectors, such as mitogen-activated protein kinase (MAPK) and yes-associated protein 1 (YAP), that contributes to accelerate abnormal cell growth (6, 7, 8, 9, 10). Therefore, these oncogenic mutants have been suggested as promising UM therapeutic targets. Recent studies determined that Gα_q/11_ inhibitors, such as FR900359, show great promise in targeting both primary and metastatic UM tumors (11, 12). However, given the central role for Gα_q/11_ in physiology, directly targeting these proteins represents a challenge. Indeed, decreasing Gα_q/11_ activity by more than 50%, as demonstrated by early gene dosage studies in mice, is likely to be lethal (13). Therefore, therapies that modulate, rather than completely abolish, Gα_q/11_ activity represent a desirable avenue. In addition to its physiological importance, high structure similarity between Gα_q/11_ and other important GTPase proteins adds another layer of complexity for selective targeting (14).

As an alternative to targeting Gα_q/11_ directly, identifying critical regulators of Gα_q/11_ may represent a promising, yet underexplored, approach. One of these is represented by regulator of G protein signaling 2 (RGS2), a member of the RGS protein superfamily, which serve as key negative regulators of G protein–mediated signal transduction (15, 16, 17). RGS2 is unique among the more than 20 known RGS proteins, in that it is a potent and selective regulator of Gα_q/11_ over other Gα subtypes (18). RGS2 is known to deactivate G_q/11_ signaling through two distinct mechanisms (18, 19, 20, 21). Through its canonical GTPase-activating protein (GAP) activity, RGS2 accelerates GTP hydrolysis at active Gα_q/11_, resulting in diminished downstream signaling. Additionally, RGS2 can act as an effector antagonist, binding active Gα_q/11_ and interrupts interactions with its downstream effectors (22). The most prevalent mutation in UM cells is the constitutively active Gα_q/11_^Q209L^, representing approximately 50% of UM cases (23). The constitutive activity for this mutant is achieved by greatly diminishing its intrinsic GTPase activity, thus RGS2 (or any other RGS protein) cannot deactivate this mutant through its canonical GAP activity (22). However, RGS2 can still act as an effector antagonist at this, and other constitutively active Gα_q/11_ mutants *in vitro* (22, 24). Therefore, we propose that targeting RGS2 is a rational, yet unexplored approach to reduce the functional abundance of oncogenic Gα_q/11_ mutants and inhibit UM tumor growth. To this end, we used UM cells, which endogenously express Gα_q_^Q209L^ (25), in the current study, as a model system to determine the effects of RGS2 on Gα_q_-dependent UM cell growth.

## Results

### RGS2 can suppress Gα_q_-dependent UM cell growth

**Figure 1:**
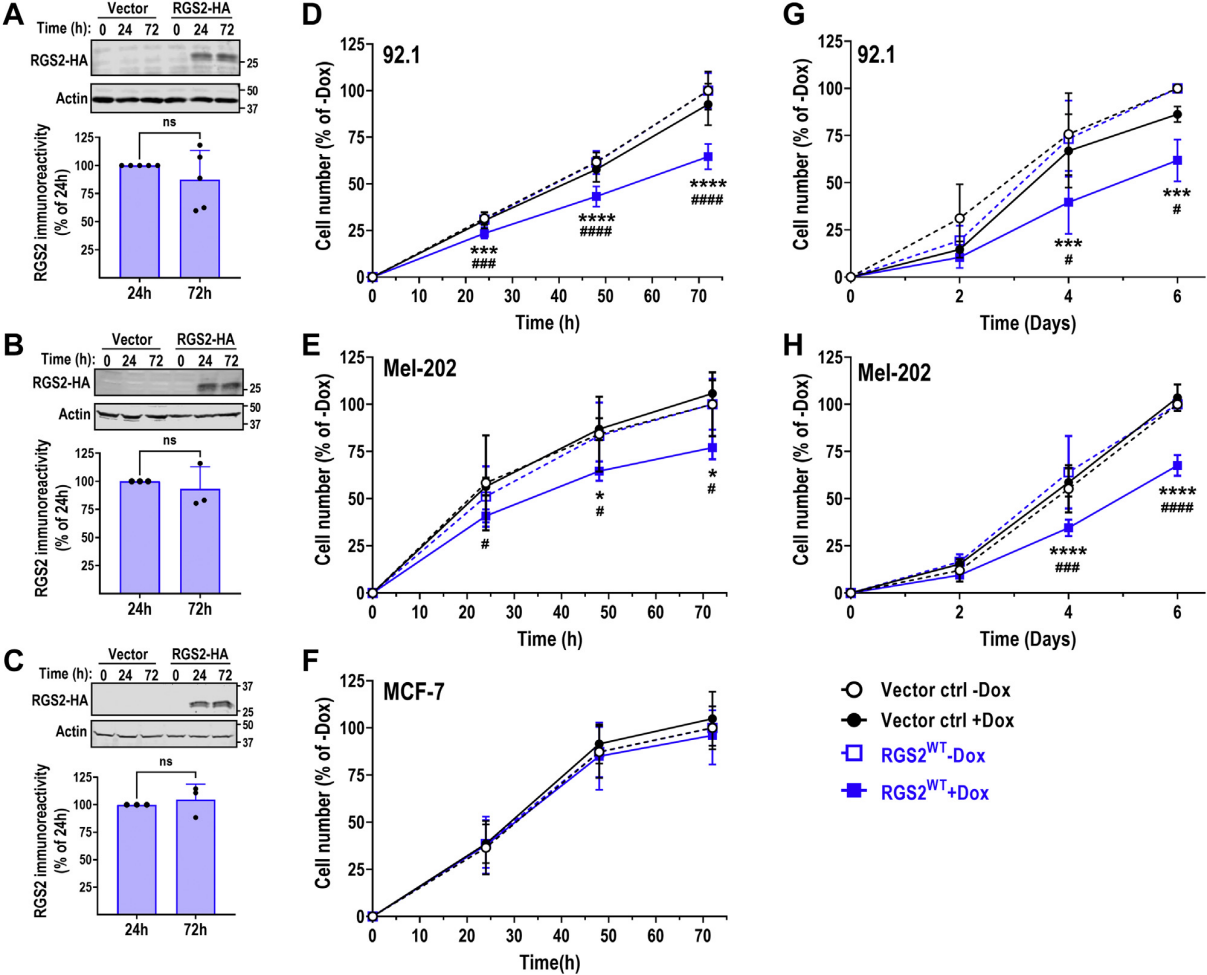
**Induction of RGS2 protein inhibits Gα**_**q**_^**Q209L**^**-dependent uveal melanoma cell growth.** Representative Western blot and quantification demonstrating reliable induction of RGS2-HA protein expression after treatment with 1 μg/ml doxycycline (Dox) in stably transduced 92.1 (*A*; N = 5), Mel-202 (*B*; N = 3), and MCF-7 (*C*; N = 3) cells. Expression levels remain stable from 24 h up to at least 72 h (nonsignificant (ns) using Student’s unpaired *t* test). Cells transduced with the empty lentivirus vector construct (Vector) served as a negative control. Cell growth of the two Gα_q_^Q209L^-dependent uveal melanoma cell lines 92.1 (*D*) and Mel-202 (*E*) is significantly reduced by induction of RGS2-HA protein expression, using continuous detection with Real-time Glo MT Cell Viability Assay up to 72 h. In contrast, Dox-induced induction of RGS2-HA had no significant effect on cell growth in the ER^+^ breast cancer cell line MCF-7 (*F*). The effect of RGS2-HA induction on cell growth of both 92.1 (*G*) and Mel-202 (*H*) cells was maintained up to 6 days, using an endpoint readout assay protocol. All data were normalized against cell growth in the absence of Dox. Data in panels *D*, *E*, *G*, and *H* are the result of five independent experiments with five technical replicates in each. Data panel *F* is the result of three independent experiments with five technical replicates in each. Cell growth after induction of RGS2-HA was compared with growth in the absence of Dox (∗) and the growth in the vector control cell line in the presence of Dox (#). ^∗/^^#^*p* < 0.05; ^∗∗∗/^^###^*p* < 0.001; ^∗∗∗∗/^^####^*p* < 0.0001 using two-way ANOVA analysis with Tukey’s *post hoc* test for pairwise comparisons. RGS, regulator of G protein signaling.

To determine whether RGS2 can suppress Gα_q_-dependent UM cell growth, we used the UM cell line 92.1, that endogenously expresses the constitutively active Gα_q_^Q209L^ mutant, as a model system. We first developed a stable cell line with doxycycline (Dox)-inducible RGS2 expression using the Lenti-Tet-One expression system (Clontech). Treatment of these cells with 1 μg/ml Dox induced expression of RGS2-HA after 24 h, which was sustained for at least 72 h (Fig. 1*A*). We then performed cell growth assays using the RealTime-Glo MT Cell Viability Assay (Promega). This nonlytic assay has the benefit of enabling multiple viability measures over time, thus enabling one well to be measured at every time point, reducing variability. We used cells stably transduced with the empty vector (vector ctrl) as a negative control. In the vector ctrl cells, Dox had no significant effect on cell growth. However, we observed a significant reduction of cell growth in cells with induced RGS2-HA expression (Fig. 1*D*), both as compared to the same cells without Dox, as well as to the vector control cells in the presence of Dox. Cell number was significantly reduced at all time points (24, 48, and 72 h), indicating that increased RGS2 expression can reduce growth of 92.1 UM cells. We used an additional UM cell line, Mel-202, which also expresses Gα_q_^Q209L^ as the oncogenic driver and created stable, Dox-inducible RGS2 protein expression in these cells as well. Similar to 92.1 cells, induction of RGS2-HA expression was induced by 1 μg/ml Dox at 24 h and remained stable for at least 72 h posttreatment (Fig. 1*B*). The induction of RGS2-HA expression resulted in a significant decrease in Mel-202 cell growth at all time points (24, 48, and 72 h), as measured by the RealTime-Glo MT Cell Viability Assay (Fig. 1*E*). In contrast, induction of RGS2-HA expression resulted in no suppression of cell growth in the breast cancer cell line MCF-7 (Fig. 1, *C* and *F*), which is not dependent on mutations in Gα_q_ as the oncogenic driver. As stated above, the chosen assay paradigm has the benefit of enabling the same well being measured at every time point; however, the reagent is only stable in cell culture for 72 h. To make sure that the effect of RGS2-HA induction on UM cell growth will not be compensated easily and quickly over time, we used an end-point approach, where cells were plated at equal cell densities on day 0, harvested on day 2, 4, and 6, respectively, and then subjected to the RealTime-Glo MT Cell Viability Assay. Dox was added every 2 days to maintain RGS2-HA induction. Using this approach, we found that the suppression of cell growth induced by RGS2-HA was maintained at least up to 6 days in both 92.1 (Fig. 1*G*) and Mel-202 (Fig. 1*H*) cells. Cell growth was significantly lower in both cell lines as compared to vector ctrl cells treated with Dox, as well as RGS2-HA transduced cells in the absence of Dox. Altogether these data suggest that RGS2 can suppress Gq-dependent UM cell growth.

### RGS2 suppression of UM cell growth is dependent on Gα_q_

**Figure 2:**
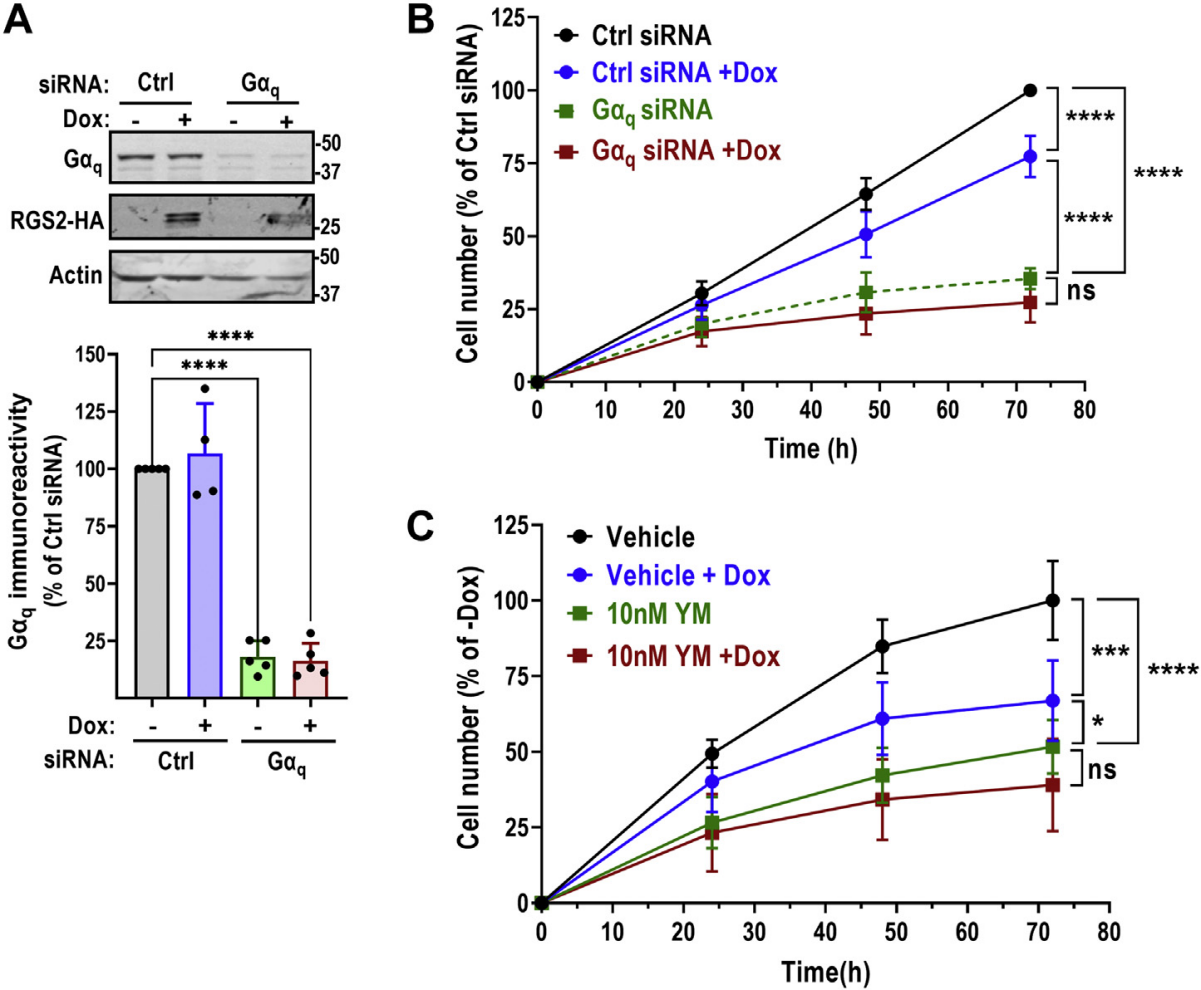
**RGS2-mediated suppression of uveal melanoma cell growth is dependent on Gα_q_.***A*, representative Western blot and quantification of five independent experiments demonstrating efficiency of siRNA-mediated knockdown of Gα_q_ in 92.1 cells and Dox induction of RGS2-HA protein expression. Knockdown efficiency was consistently ∼80%. ^∗∗∗∗^*p* < 0.0001 using one-way ANOVA with Tukey’s *post hoc* test for pairwise comparisons. *B*, cell growth of 92.1 cells was significantly inhibited by induction of RGS2-HA protein expression or Gα_q_ siRNA. siRNA-mediated knockdown of Gα_q_ caused a significantly greater decrease in cell growth than RGS2 protein induction. In addition, the effect of RGS2 on cell growth was lost when Gα_q_ was concurrently knocked down. Shown here is the result of five independent experiments with five technical replicates in each. *C*, cell growth of 92.1 cells was significantly inhibited by induction of RGS2-HA protein expression or the Gα_q_ inhibitor YM-254890 (10 nM). Similar to siRNA-mediated Gα_q_ knockdown, YM-254890 treatment caused a significantly greater decrease in cell growth than RGS2 protein induction. In addition, the effect of RGS2 on cell growth was lost in the presence of YM-254890. Result of three independent experiments with five technical replicates in each. ^∗^*p* < 0.05; ^∗∗∗^*p* < 0.001; ^∗∗∗∗^*p* < 0.0001 using two-way ANOVA with Tukey’s *post hoc* test for pairwise comparisons. RGS, regulator of G protein signaling.

After establishing that RGS2 can inhibit Gα_q_^Q209L^-dependent UM cell growth, we next aimed to determine whether this suppression was mediated through actions of RGS2 on Gα_q_. siRNA-mediated knockdown of Gα_q_ in our stable 92.1 cell line resulted in an 83% reduction in Gα_q_ protein levels, as detected by Western blot (Fig. 2*A*). This resulted in a large decrease in 92.1 UM cell growth (Fig. 2*B*), indicating that Gα_q_ is a major driver of proliferation in these cells, in agreement with previous literature (25). To validate the SMART-POOL siRNA used in these studies, we used the individual siRNA oligos. All four individual siRNAs resulted in a significant reduction in both Gα_q_ protein levels (Fig. S1, *A* and *B*) and 92.1 cell growth (Fig. S1*C*), although to lesser extent than what was achieved by the SMART-POOL siRNA. Dox-mediated induction of RGS2-HA in cells transfected with a nontargeting siRNA control (ctrl siRNA) resulted in significant reduction in cell growth (Fig. 2*B*), at a similar magnitude as we had initially observed (Fig. 1). However, RGS2 lost the ability to reduce cell growth in cells transfected with Gα_q_ siRNA, indicating that RGS2 suppresses 92.1 UM cell growth through actions that are dependent on Gα_q_. To complement our genetic approach to inhibit Gα_q_, we used YM-254890 a selective Gα_q/11_ inhibitor (26). In agreement with previous studies, YM-254890 caused a significant decrease in 92.1 cell growth (Figs. 2*C* and S1*D*) (27). In the presence of YM-254890, induction of RGS2-HA expression had no additional effect on 92.1 cell growth (Fig. 2*C*), confirming that RGS2 suppresses Gq-dependent UM cell growth through a mechanism that is dependent on the presence and activity of Gα_q_.

### RGS2 suppression of UM cell growth is mediated through association with Gα_q_

**Figure 3:**
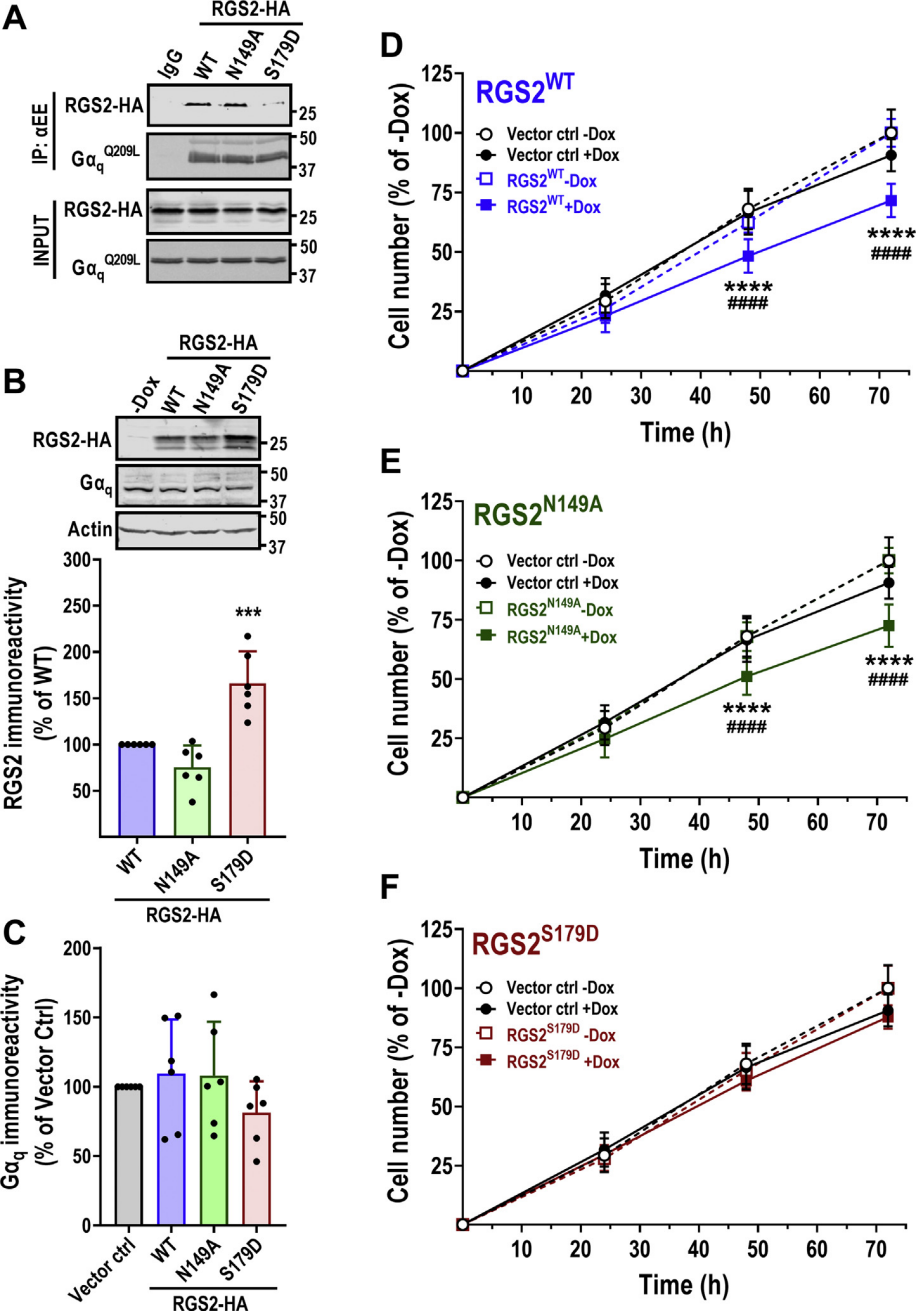
**RGS2-mediated suppression of uveal melanoma cell growth is dependent on association with Gα**_**q**_^**Q209L**^**.***A*, Gα_q_^Q209L^, RGS2^WT^, RGS2^N149A^ (GAP deficient), and RGS2^S179D^ were expressed by *in vitro* transcription translation and subjected to co-IP, utilizing a Glu-Glu (EE) tag on Gα_q_. Both RGS2^WT^ and RGS2^N149A^ associated with Gα_q_^Q209L^, while RGS2^S179D^ did not. Representative Western blot (*B*) and quantification of six independent experiments (*C*) demonstrating induction of RGS2^WT^-HA, RGS2^N149A^-HA, and RGS2^S179D^-HA protein expression after treatment with 1 μg/ml Dox in stably transduced 92.1 cells. RGS2^S179D^ expressed at significantly higher levels than RGS2^WT^ or RGS2^N149A^. *D*, in the same experiments, induction of RGS2-HA protein expression did not affect protein expression levels of Gα_q_. ^∗∗∗^*p* < 0.001 using one-way ANOVA with Tukey’s *post hoc* test for pairwise comparisons. Cell growth of 92.1 cells was inhibited by induction of RGS2^WT^ (*D*) and RGS2^N149A^ (*E*), but not by RGS2^S179D^ (*F*), despite this mutant displaying higher protein levels. Data were normalized against cell growth in the absence of Dox. Result of three independent experiments with five technical replicates in each. Cell growth after induction of RGS2-HA was compared with growth in the absence of Dox (∗) and the growth in the vector control cell line in the presence of Dox (#). ^∗∗∗∗/^^####^*p* < 0.0001 using two-way ANOVA analysis with Tukey’s *post hoc* test for pairwise comparisons. Dox, doxycycline; RGS, regulator of G protein signaling.

We next aimed to determine the mechanism by which RGS2 suppresses Gα_q_^Q209L^-mediated cell growth in 92.1 cells. Previous studies have shown that while RGS2 does not have GAP activity toward Gα_q_^Q209L^, it can still bind to this constitutively active mutant (22, 28). To confirm this, we used RGS2^N149A^, which is thought to possess no GAP activity toward any Gα subtype (29, 30). In addition, we used RGS2^S179D^, which has previously been shown to have impaired binding to Gα_q_ (31). We first confirmed the ability for RGS2^WT^, RGS2^N149A^, and RGS2^S179D^ to bind Gα_q_^Q209L^ using proteins expressed by *in vitro* transcription/translation, followed by co-immunoprecipitation (co-IP) utilizing a Glu-Glu (EE) tag on Gα_q_^Q209L^. Both RGS2^WT^ and RGS2^N149A^ associated with Gα_q_^Q209L^ to a similar degree (Fig. 3*A*). In contrast, RGS2^S179D^ was severely deficient in associating with Gα_q_^Q209L^ (Fig. 3*A*). Next, we determined the effect of RGS2^N149A^ and RGS2^S179D^ on 92.1 cell growth. We created stable 92.1 cell lines with Dox-inducible expression of either mutant using the same strategy as for RGS2^WT^. Following induction with 1 μg/ml Dox, RGS2^N149A^ expressed at similar levels as RGS2^WT^. In contrast, RGS2^S179D^ was expressed at significantly higher levels than RGS2^WT^ (Fig. 3*B*). Gα_q_ protein levels were unchanged by induction of either RGS2 variant (Fig. 3*C*). We next determined the effect of the GAP-deficient RGS2^N149A^ and the Gα_q_ binding-deficient RGS2^S179D^ on 92.1 cell growth. Similar to what we had observed with RGS2^WT^ (Figs. 1 and 3*D*), RGS2^N149A^ significantly inhibited cell growth (Fig. 3*E*), indicating that RGS2 inhibits Gα_q_-dependent UM cell growth through a GAP-independent mechanism. In contrast. Induction of RGS2^S179D^ did not significantly change 92.1 cell growth (Fig. 3*F*), indicating that RGS2 suppresses UM cell growth through a mechanism that involves binding to Gα_q_. The suppression of cell growth by RGS2^WT^ and RGS2^N149A^ were significantly decreased at 48 and 72 h. Altogether our data support a model where RGS2 inhibits Gα_q_^Q209L^-mediated cell proliferation through effector antagonism, rather than through its canonical GAP activity.

### RGS2 suppresses Gα_q_-mediated extracellular signal-regulated kinase 1/2 phosphorylation in Gq-dependent UM cells

**Figure 4:**
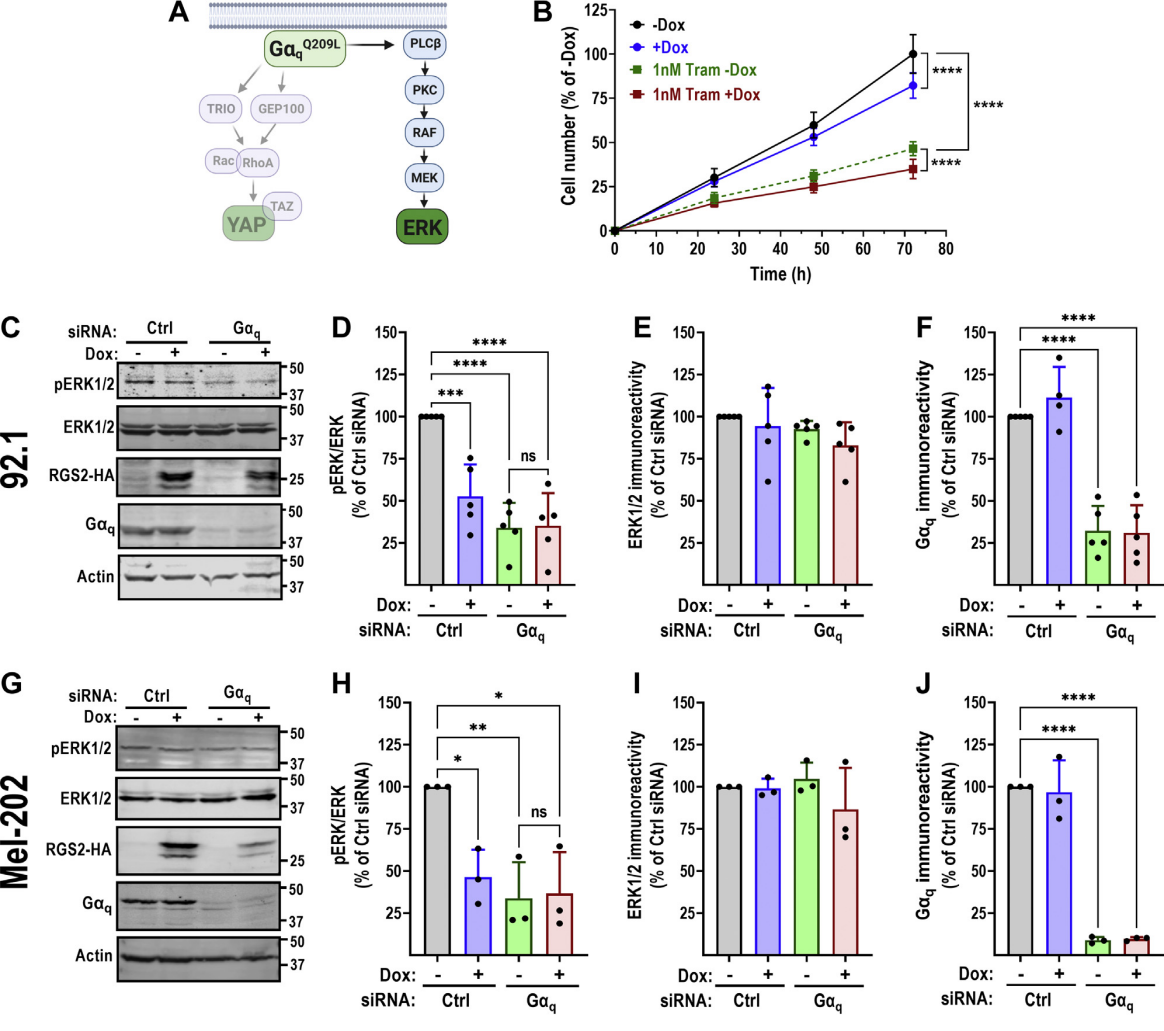
**RGS2 inhibits Gα**_**q**_**-mediated ERK1/2 phosphorylation in uveal melanoma cells.***A*, schematic describing the signaling pathway leading to ERK1/2 phosphorylation downstream of Gα_q_. *B*, the MEK1/2 inhibitor trametinib (Tram) inhibits 92.1 cell growth. Induction of RGS2-HA with Dox enhances the effect of Tram only at low concentrations (≤1 nM; and see Fig. S2). Result of three independent experiments with five technical replicates in each. ∗∗∗∗*p* < 0.0001 using two-way ANOVA with Tukey’s *post hoc* test for pairwise comparisons. Representative Western blot (*C*) and quantification of five independent experiments (*D*) demonstrating that both RGS2-HA induction and siRNA-mediated Gα_q_ knockdown results in significantly reduced levels of ERK1/2 phosphorylation in 92.1 cells. *E*, protein levels of total ERK1/2 are unaffected by either Dox or Gα_q_ siRNA. *F*, efficiency of siRNA-mediated Gα_q_ knockdown in 92.1 cells was ∼70%. Representative Western blot (*G*) and quantification of three independent experiments (*H*) demonstrating that both RGS2-HA induction and siRNA-mediated Gα_q_ knockdown results in significantly reduced levels of ERK1/2 phosphorylation in Mel-202 cells. *I*, protein levels of total ERK1/2 are unaffected by either Dox or Gα_q_ siRNA. *J*, efficiency of siRNA-mediated Gα_q_ knockdown in Mel-202 cells was ∼90%. The effect of RGS2 on ERK1/2 phosphorylation is lost when Gα_q_ was concurrently knocked down in both 92.1 and Mel-202 cells. ∗*p* < 0.05; ^∗∗^*p* < 0.01; ^∗∗∗^*p* < 0.001; ^∗∗∗∗^*p* < 0.0001 using one-way ANOVA with Tukey’s *post hoc* test for pairwise comparisons. Dox, doxycycline; ERK1/2, extracellular signal-regulated kinase 1/2; RGS, regulator of G protein signaling.

We next aimed to determine which signaling pathway(s) downstream of Gα_q_ are affected by RGS2 protein induction. Two main signaling pathways are associated with cell proliferation downstream of Gα_q_. First, Gα_q_ activates phospholipase Cβ (PLCβ), which subsequently leads to activation of protein kinase C, and downstream activation of a MAPK cascade leading to phosphorylation of extracellular signal-regulated kinase 1/2 (ERK1/2) (Figs. 4*A*). We therefore used phosphorylation of ERK1/2 as a readout of signaling downstream of Gα_q_^Q209L^ *via* the canonical PLCβ pathway. First, the MEK1/2 inhibitor trametinib (Tram) was used to determine effects of ERK1/2 activation on 92.1 cell growth. Treatment of 92.1 cells with Tram caused complete abolishment of ERK1/2 phosphorylation (data not shown) and a corresponding strong, dose-dependent inhibition of cell growth (Fig. S2). At low concentrations of Tram (≤1 nM), induction of RGS2 caused additional suppression of cell growth (Fig. 4*B*). This effect was lost at higher Tram concentrations (Fig. S2). We next used siRNA to knock down Gα_q_ in the Dox-inducible RGS2^WT^ 92.1 cells. Gα_q_ siRNA caused a significant reduction in Gα_q_ protein levels, as determined by Western blot (Fig. 4, *C* and *F*). Under basal conditions, these cells displayed a high level of ERK1/2 phosphorylation (Fig. 4, *C* and *D*). Gα_q_ siRNA caused a significant decrease in ERK1/2 phosphorylation, consistent with the model that Gα_q_ drives proliferation in these cells *via* activation of MAPK signaling (Fig. 4, *C* and *D*). Induction of RGS2-HA expression also caused a significant decrease in ERK1/2 phosphorylation (Fig. 4, *C* and *D*). However, RGS2 lost the ability to reduce ERK1/2 phosphorylation when Gα_q_ was concurrently knocked down. Total ERK1/2 protein levels were unaffected by either Dox or Gα_q_ siRNA (Fig. 4, *C* and *E*). The effects of RGS2-HA induction and Gα_q_ knockdown on ERK1/2 phosphorylation could be replicated in a second Gα_q_^Q209L^-dependent UM cell line, Mel-202 (Fig. 4, *G*–*J*). Importantly, medium to high concentrations of Tram caused complete abolishment of ERK1/2 phosphorylation (data not shown), with virtually complete arrest in cell growth as a result (Fig. S2). In contrast, both Dox-induced induction of RGS2 protein or Gα_q_ siRNA only caused partial inhibition (50%–70%) of ERK1/2 phosphorylation. Altogether these data are consistent with the model that RGS2 mediates effects on ERK1/2 signaling through actions that are dependent on Gα_q_.

**Figure 5:**
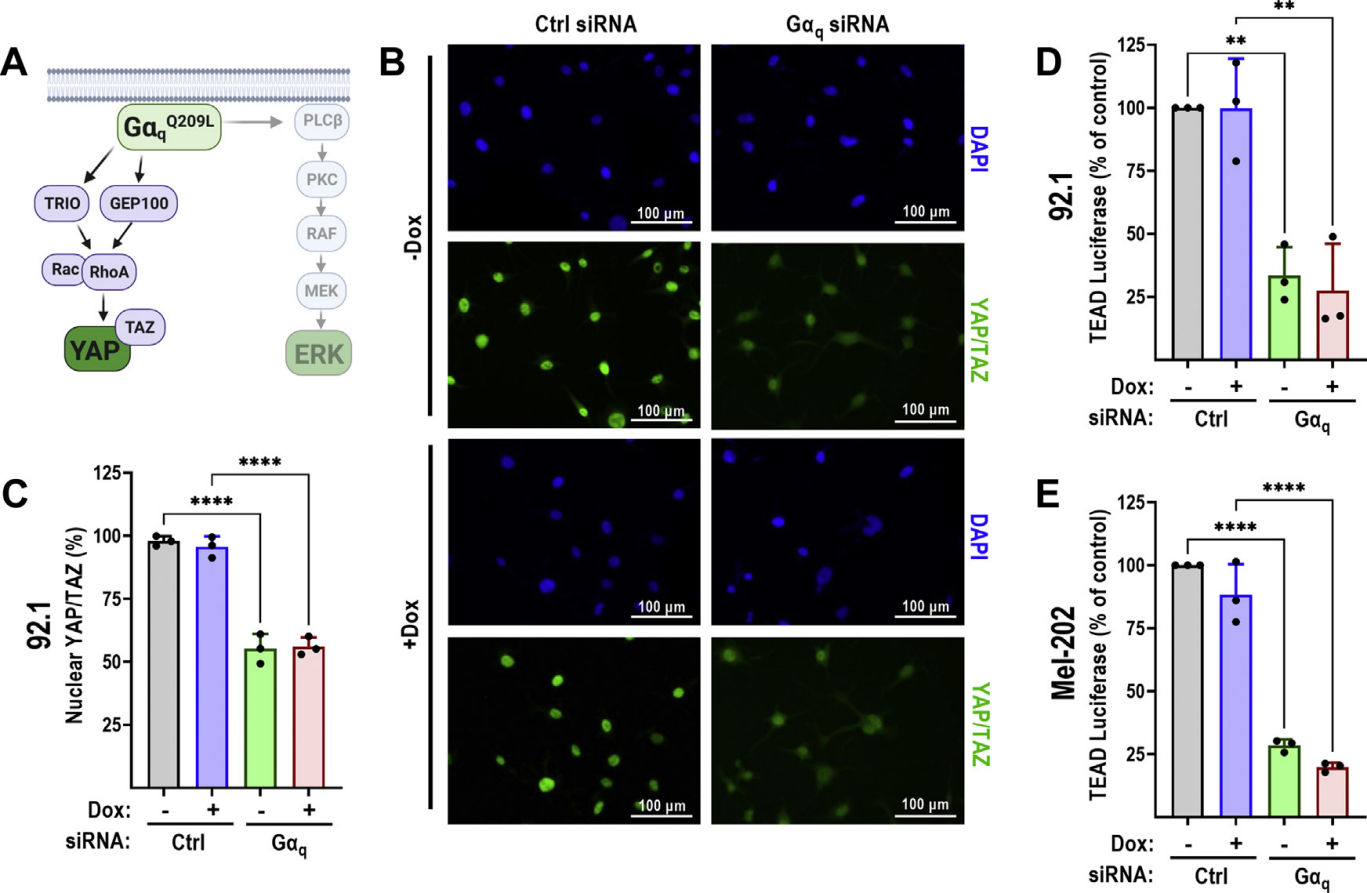
**RGS2 does not affect Gα**_**q**_**-mediated YAP activation in uveal melanoma cells.***A*, schematic describing the signaling pathway leading to YAP activation downstream of Gα_q_. Representative images (*B*) and quantification (*C*) of three independent experiments demonstrating effects of Gα_q_ siRNA and Dox-induced RGS2 expression on nuclear localization of YAP/TAZ. Gα_q_ siRNA results in almost a 50% reduction in YAP/TAZ nuclear localization. Induction of RGS2 protein expression has no effect on YAP/TAZ nuclear localization. YAP/TAZ activation was measured using a lentiviral TEAD luciferase reporter in 92.1 (*D*) and Mel-202 (*E*) cells. In both cell lines, Gα_q_ siRNA results in a significant (∼75%) reduction in TEAD luciferase activity. Induction of RGS2-HA has no effect on TEAD luciferase. Results of three independent experiments in both 92.1 and Mel-202 cells. ^∗∗^*p* < 0.01; ^∗∗∗∗^*p* < 0.0001 using one-way ANOVA with Tukey’s *post hoc* test for pairwise comparisons. Dox, doxycycline; RGS, regulator of G protein signaling; YAP, yes-associated protein 1.

**Figure 6:**
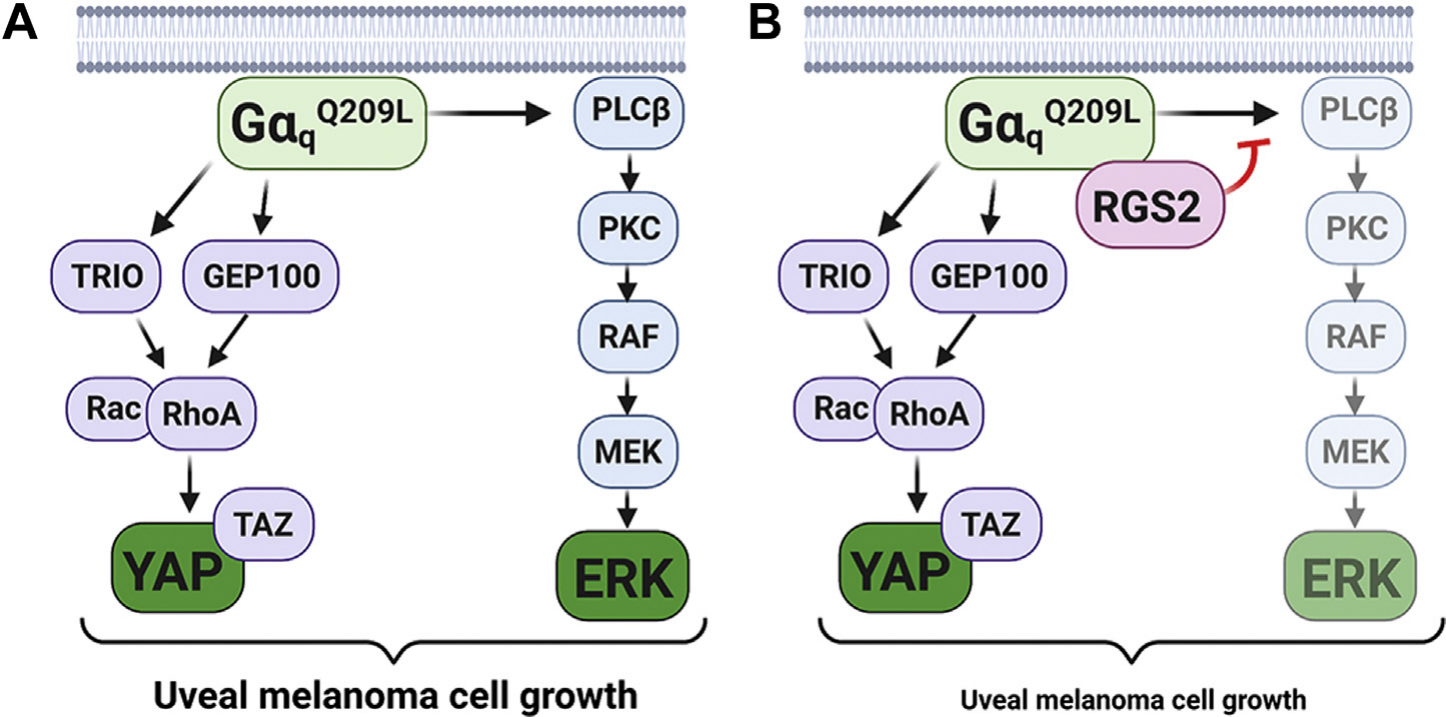
**Model for RGS2 effects on Gα**_**q**_**-dependent uveal melanoma cell growth.***A*, Gα_q_^Q209L^ drives 92.1 uveal melanoma cell growth through activation of both YAP/TAZ and ERK1/2. *B*, RGS2 inhibits 92.1 uveal melanoma cell growth by binding Gα_q_^Q209L^ and acting as an effector antagonist for ERK1/2 activation only, while leaving YAP/TAZ activation unaffected. This results in only partial inhibition of Gα_q_^Q209L^ induced cell growth. ERK1/2, extracellular signal-regulated kinase 1/2; RGS, regulator of G protein signaling; YAP, yes-associated protein 1.

### RGS2 does not affect Gα_q_-mediated activation of YAP in UM cells

The other main signaling pathway that connects Gα_q_ to cell proliferation in UM is through constitutive activation and nuclear localization of YAP/TAZ, enabling transcription of genes that promote cell growth (7, 10, 23, 32). This signaling is independent of PLCβ and the canonical Hippo pathway and mediated instead through a Trio-Rho/Rac signaling mechanism (7) (Figs. 5*A* and 6*A*). We therefore next aimed to determine the effects of RGS2 on Gα_q_^Q209L^-mediated YAP/TAZ activation in Gq-dependent UM cell lines. First, we measured nuclear YAP/TAZ localization in 92.1 cells, using fluorescence microscopy. We observed that YAP/TAZ were constitutively nuclear even at densities with high cell–cell contact which would normally drive YAP/TAZ out of the nucleus, in nontransformed, primary cells (33, 34) (Fig. 5, *B* and *C*). Once restricted to the cytosol, YAP/TAZ are constitutively degraded (35), resulting in a decrease in fluorescence in our assay system. Consistent with previous observations (10), siRNA-mediated knockdown of Gα_q_ reduced nuclear YAP/TAZ localization as measured by a reduction in fluorescence signal (Fig. 5, *B* and *C*). However, in contrast to what was observed in our ERK1/2 phosphorylation experiments, Dox-induced expression of RGS2 had no effect on YAP/TAZ nuclear localization (Fig. 5, *B* and *C*). This lack of effect was independent of whether Gα_q_ was knocked down with siRNA (Fig. 5, *B* and *C*).

As a direct measure of YAP/TAZ activity, we next used a TEAD luciferase reporter in both 92.1 and Mel-202 cells, as a measure of YAP/TAZ transcriptional activity. At baseline, both UM cell lines showed high levels of TEAD luciferase activity, and siRNA-mediated Gα_q_ knockdown significantly reduced luciferase signal in both cell lines (Fig. 5, *D* and *E*). Again, induction of RGS2-HA had no effect on TEAD luciferase signal, regardless of whether Gα_q_ was knocked down or not (Fig. 5, *D* and *E*), providing further support that RGS2 cannot inhibit YAP/TAZ signaling downstream of Gαq in these UM cell lines.

Altogether, our data indicate that RGS2 can partially suppress Gα_q_^Q209L^-dependent UM cell growth, through an effector antagonism mechanism (Fig. 6). Gα_q_ promotes UM cell growth through two main signaling pathways; however, RGS2 can only suppress the MAPK kinase cascade, while leaving the YAP/TAZ signaling unaffected.

## Discussion

RGS proteins have emerged in recent years as key regulators of G protein–mediated signaling, through both their canonical GAP activity, as well as GAP-independent mechanisms. RGS2 is unique in its selectivity for Gα_q_ over other Gα subtypes. This selectivity applies not only to GAP activity but also in its previously identified role as an effector antagonist (22). In the current study, we identified a novel role for RGS2 in suppressing Gα_q_^Q209L^-mediated UM cell growth, independent of its canonical GAP activity. This adds another layer of complexity for the actions of RGS2 on Gq-mediated signaling pathways.

We used both 92.1 and Mel-202 cells, which both harbor the Gα_q_^Q209L^ mutation. While this is the most prevalent mutation in UM, other mutations in both Gα_q_ and Gα_11_ represent a large fraction of UM cases. Further studies are needed to determine the effects of RGS2 on these mutations as well. Gα_q_^Q209P^ is the second most common mutation. A previous study determined that this mutant adopts a conformation different from that of Gα_q_^Q209L^ or active Gα_q_^WT^, causing altered binding to effectors, including RGS proteins (36). Thus, further studies are warranted to determine whether RGS2 can inhibit UM cell growth mediated by other Gα_q/11_ activating mutations.

Throughout the current study, induction of RGS2 protein expression consistently resulted in ∼20% reduction in UM cell growth. This relatively small reduction was due to RGS2 only inhibiting Gq-mediated ERK1/2 phosphorylation, while leaving YAP/TAZ activation unaffected. Complete abolishment of ERK1/2 phosphorylation induced by the clinically used MEK1/2 inhibitor Tram, and the resulting arrest of 92.1 cell growth (Fig. S2) indicates that abolishing ERK1/2 phosphorylation is enough to completely halt 92.1 cell growth. However, RGS2 induction, like Gα_q_ knockdown, did not completely abolish ERK1/2 phosphorylation (Fig. 4), explaining the difference in cell growth responses compared to Tram. The partial inhibition of ERK1/2 phosphorylation by RGS2 induction is not surprising, given that RGS2 acts on this pathway through actions on Gα_q_. Therefore, it is not possible for RGS2 induction to cause a larger effect than that of knocking down Gα_q_ in these cells. Instead, the larger effect of knocking down Gα_q_ on UM cell growth can be explained by partial inhibition of two pathways, both contributing to UM cell growth (Fig. 6).

The clinical relevance of this relatively small effect of RGS2 is yet to be determined, as is the molecular determination of how RGS2 interacts with Gα_q_ to only affect the PLCβ ‘signaling arm’. In 92.1 cells, we observed constitutively nuclear YAP/TAZ even at densities with high cell–cell contact which would normally drive YAP/TAZ out of the nucleus. This could possibly explain why RGS2 is not able to cause YAP/TAZ nuclear exclusion, as the modulatory effect of RGS2 on Gα_q_ is not enough to achieve this. The relatively modest effect we observed on UM cell growth by RGS2 may not be detrimental. In fact, from a clinical standpoint, and as eluded to in the Introduction, modulation, rather than abolishment of Gα_q_ activity, may provide a safer alternative for therapeutic development. Indeed, decreasing Gα_q/11_ activity by more than 50%, as demonstrated by early gene dosage studies in mice, is likely to be lethal (13). Thus, the ∼20% reduction in cell growth observed here may be enough to have therapeutic effect. In addition, we previously determined that increasing RGS2 protein levels as little as 2-fold is enough to have functional effects on GPCR signaling both *in vitro* and *in vivo* (37, 38). Thus, modest increases in RGS2 protein function could have substantial beneficial effects also in UM driven by constitutively active Gα_q_. While more studies are needed to determine the effect of RGS2 on UM driven by mutations other than Gα_q_^Q209L^, our current study provides evidence that RGS2 could be a promising avenue to pursue for future targeted therapies in UM.

## Experimental procedures

### Materials

All chemicals and reagents were purchased from Thermo Fisher Scientific unless otherwise stated. YM-254890 (#AG-CN2-0509-MC05) was from Adipogen Life Sciences. Tram was from Selleck Chemicals.

### DNA constructs

RGS2-HA (in pcDNA3.1) was from cDNA resource center. RGS2^N149A^ and RGS2^S179D^ were created from the RGS2-HA (in pcDNA3.1) construct using QuikChange II site-directed mutagenesis (Agilent Technologies) according to the manufacturer’s instructions. Each was cloned into pF3K WG (BYDV) Flexi (Promega), and pLVX-TetOne-Puro (Clontech/Takara Bio), respectively. pLVX-TetOne-Puro and related materials were kindly provided by Dr Michael Wendt, Purdue University. GNAQ (in pcDNA3.1) was from cDNA resource center. GNAQ^Q209L^ was created from GNAQ using QuikChange II site-directed mutagenesis. Each was cloned into pF3K WG (BYDV) Flexi. The Primer sequences for construct generation and mutagenesis are available by request.

### Antibodies

Rabbit anti-HA (H6908), mouse anti-β-Actin (A2228), and rabbit anti-β-Actin (A2066) were from Millipore Sigma. Mouse anti-Gα_q_ (sc-136181) was from Santa Cruz. Mouse anti-Glu-Glu tag (901801) was from Biolegend. Mouse anti-pERK (9106S), rabbit anti-ERK1/2 (4695S), and rabbit anti-YAP/TAZ (D24E4) were from Cell Signaling. Western blot secondary IRDye 800CW and 680RD antibodies were all from Li-Cor Biosciences. Immunocytochemistry secondary antibody goat anti-rabbit, Alexa flour 488 (A-11008) was purchased from Invitrogen.

### Cell culture

Cells were maintained in a humidified incubator at 37 °C with 5% CO_2_. Human UM 92.1 cells (Millipore Sigma, #13012458) and Mel-202 cells (Millipore Sigma, #13012457) were grown in Roswell Park Memorial Institute 1640 medium (RPMI 1640; Gibco, #11875-093), supplemented with 10% fetal bovine serum (Gibco, #16000), 100 U/ml penicillin/100 μg/ml streptomycin (Gibco, #15140). Human embryonic kidney 293T (HEK-293T) cells and MCF-7 breast cancer cells (ATCC, #HTB-22) were grown in Dulbecco’s modified Eagle’s medium (Gibco, #11995), supplemented with 10% fetal bovine serum, 100 U/ml penicillin/100 μg/ml streptomycin.

### Stable cell line development

Lentiviral particles containing either RGS2^WT^-HA, RGS2^N149A^-HA, or RGS2^S179D^-HA (in pLVX-TetOne-Puro) were produced in HEK-293T cells and used to transduce 92.1, Mel-202 and MCF-7 cells. Stable integration was selected using 1 μg/ml puromycin (Fisher Scientific) for 2 weeks. The stable cell lines were induced with Dox (Fisher Scientific) at 1 μg/ml for 24 to 72 h (as indicated in figure legends), and successful induction of RGS2 protein expression was detected by Western blot as described below.

### siRNA transfections

siRNA transfection was performed under reduced serum conditions (Opti-MEM) 24 h prior to transfections at 40% to 60% confluency. Cells were transfected with siGENOME SMART-POOL siRNA (Gq, M-008562-00-0005 and nontargeting, D-001206-13-05) from Dharmacon using Lipofectamine RNAiMAX (Thermo Fisher/Invitrogen). Sequences for each siRNA is described in Table S1. Western blot was performed to detect knockdown efficiency.

### Preparation of cell lysates

Cells were harvested on ice in lysis buffer containing protease inhibitors (20 mM Tris-HCl (pH 7.4), 150 mM NaCl, 1 mM EDTA, 1 mM β-glycerophosphate, 1% Triton X-100, 0.1% SDS, and cOmplete Protease Inhibitor Cocktail EDTA-free [Roche]). Lysates were sonicated for 10 min at 4 °C, centrifuged at 14,800 rpm for 10 min, and the supernatants were used for SDS-PAGE and Western blot. Total protein concentration was determined using the Pierce BCA Protein Assay Kit (Thermo Scientific).

### *In vitro* transcription/translation and co-IP

TnT SP6 High-Yield Wheat Germ Protein Expression System kit (L3260; Promega) was used as directed by the manufacturer. The transcription/translation reaction was performed using 2 μg DNA (RGS2 or GNAQ in pF3K WG (BYDV) Flexi) for 2 h at room temperature. Translated proteins were mixed and allowed to form complexes for 1 h at room temperature with gentle rotation in RIPA buffer (50 mM Tris-HCl [pH 7.4], 150 mM NaCl, 0.25% w/v Deoxycholate, 1 mM EDTA, 1% NP-40, complete Protease Inhibitor Cocktail EDTA-free). 30 μl was removed saved as input. 2 μl mouse anti-Glu-Glu antibody was added, and co-IP was allowed to proceed for 16 to 18 h at 4 °C with gentle rotation. 20 μl Protein G agarose beads (Roche) was added, followed by 2 h incubation at 4 °C with gentle rotation. Samples were washed three times with RIPA buffer and once with phosphate buffered saline. Bound proteins were eluted at 95 °C in 30 μl SDS sample buffer (Li-Cor Biosciences).

### SDS-PAGE and Western blot

Equal amounts of protein in each lane were resolved on a 10% to 12% SDS-PAGE gel for 1 h at 160 V. Samples were transferred to an Immobilon-FL PVDF membrane (Millipore) and subjected to Western immunoblot analysis using Li-Cor blocking buffer for both blocking and antibody diluents. Membranes were blocked for 1 h, then incubated 16 to 18 h in primary antibodies, and 1 h with IRDye secondary antibodies. Following each antibody incubation, membranes were washed four times in phosphate buffered saline with 0.1% Tween-20. Membranes were imaged using a Li-Cor Odyssey CLx imager (Li-Cor Biosciences).

### Real-time Glo cell growth assay

Real-time Glo MT Cell Viability Assay Kit (G9711, Promega) was used as instructed. For cell growth assay detected in real-time, cells were plated at low density (50–150 cells/well) in 20 μl complete culture media, in a white 384-well plate (CulturPlate-384, #6007680, PerkinElmer). 20 μl 2× MT substrate and NanoLuc enzyme with or without Dox (final concentration 1 μg/ml) was added, and plates were centrifuged at 1000 rpm for 1 min to collect cells and reagents at the bottom of each well. Plates were incubated at 37 °C and luminescence was detected at 1 h (baseline), 24 h, 48 h, and 72 h on a Synergy Neo2 plate reader (BioTek) using the same gain (continuous protocol with reads from the same well at every time point). For 6-days cell growth assays (end-point protocol), cells were plated at equal cell densities (5000–10,000 cells/well) in a 24-well plate in 500 μl complete culture media. On the second day, cells were collected (baseline, day 0), diluted 20× in culture media, and re-plated in 20 μl complete culture media in a white 384-well plate. 20 μl 2× MT substrate and NanoLuc enzyme were added, followed by 1 h incubation at 37 °C, and luminescence detected using a Synergy Neo2 plate reader. This procedure was repeated on day 2, 4, and 6. Fresh Dox was added ever 48 h to maintain RGS2-HA expression.

### Immunofluorescence imaging

Cells were plated into tissue culture treated 96-well plates and allowed to attach for 6 h siRNA transfection and Dox treatment was carried out as described above. Cells were fixed in 4% paraformaldehyde, permeabilized with 0.25% Triton X-100, blocked with 1% bovine serum albumin, and incubated with YAP/TAZ primary antibody 1:200 overnight at 4 °C, and anti-rabbit secondary antibody 1:1000 for 1 h at room temperature, cells were imaged, and YAP/TAZ nuclear localization was quantified as previously described (39, 40, 41) using a Cytation5 (Biotek). Briefly, cell nuclei were identified using the DAPI channel and mean YAP/TAZ nuclear intensity was determined for the control siRNA, Dox group. A threshold value of 85% of this intensity was then used to quantify the percentage of YAP/TAZ nuclear positive cells after treatments. In initial experiments developing this image analysis protocol, we compared the automated software analysis to two different investigators asked to identify nuclear positive YAP/TAZ cell populations, blinded to the conditions. The automated analysis was within 5% variance of the two investigators, for each condition.

### Luciferase reporter assay

Cells were plated at 50% to 60% density and incubated at 37 °C overnight. Cells were transfected with siRNA using the protocol described above. After 4 h, Opti-MEM was replaced with normal cell culture medium, containing TEAD-Luc lentivirus particles (#79833, BPS Bioscience), 5 μg/ml Polybrene (#sc-134220, Santa Cruz Biotechnology), and 1 μg/ml Dox. Transduction was allowed to proceed at 37 °C for 16 to 18 h. 2000 cells were re-plated in a 384 well plate using serum-free medium. Gly-Phe-7-Amino-4-trifluoromethylcoumarin (GF-AFC) viability substrate (MP Biomedicals) was diluted 1:1000 in serum-free medium, and 5 μl was added to each well. Plates were incubated at 37 °C for 30 min, then fluorescence was detected on a Synergy Neo2 multimode plate reader (BioTek). 15ul luciferase substrate assay solution (E2610, Promega) was added, followed by 5 min incubation at room temp and detection of luminescence on a Synergy Neo2 plate reader.

### Statistical analysis

Western blot images were captured and quantified using Image Studio software (Li-Cor Biosciences). For western blots, band intensity for proteins were normalized against a loading control. pERK1/2 was normalized against total ERK1/2. All data were analyzed using GraphPad Prism 9.0 (GraphPad). Datasets with two groups were analyzed using Student’s unpaired *t* test. Datasets with three or more groups were analyzed with one-way or two-way ANOVA, depending on the nature of the groups as indicated in each figure. Groups were compared with Tukey’s *post hoc* test for multiple comparisons. All experiments were run at least three times. Data are presented as mean ± SEM with a *p*-value less than 0.05 considered significant.

## Data availability

All available data are contained within this manuscript. Sequences for primers, siRNA, and constructs are available upon request. DNA constructs are available upon request. These should be made to jsjogren@purdue.edu.

## Supporting information

This article contains supporting information.

## Conflicts of interest

The authors declare that they have no conflicts of interest with the contents of this article.
