# Supplementary material for: Regulator of G protein signaling 2 inhibits Gαq-dependent uveal melanoma cell growth
Source: J Biol Chem. 2022 Apr 19;298(6):101955. doi: 10.1016/j.jbc.2022.101955 (PMC9120238; doi:10.1016/j.jbc.2022.101955)
Supplement: Supplemental Figures S1, S2 and Table S1 [file mmc1.pdf]

SUPPORTING INFORMATION

Regulator of G protein signaling 2 inhibits Gα$_q$-dependent uveal melanoma cell growth
Qian Zhang, Andrew J. Haak and Benita Sjögren

Supplemental Tables

Table S1. Sequences of individual GNAQ siRNA oligos in the siGENOME SMART-POOL (Dharmacon M-008562-00-0005).

siRNA	Dharmacon cat#	Sequence
siGNAQ-1	D-008562-01	CAAUAAGGCUCAUGCACAA
siGNAQ-2	D-008562-02	GCAACAAGAUGUGCUUAGA
siGNAQ-3	D-008562-03	GCAAGGCUCUCUUUAGAAC
siGNAQ-4	D-008562-04	UAGUAGCGCUUAGUGAAUA

Supplemental Figures

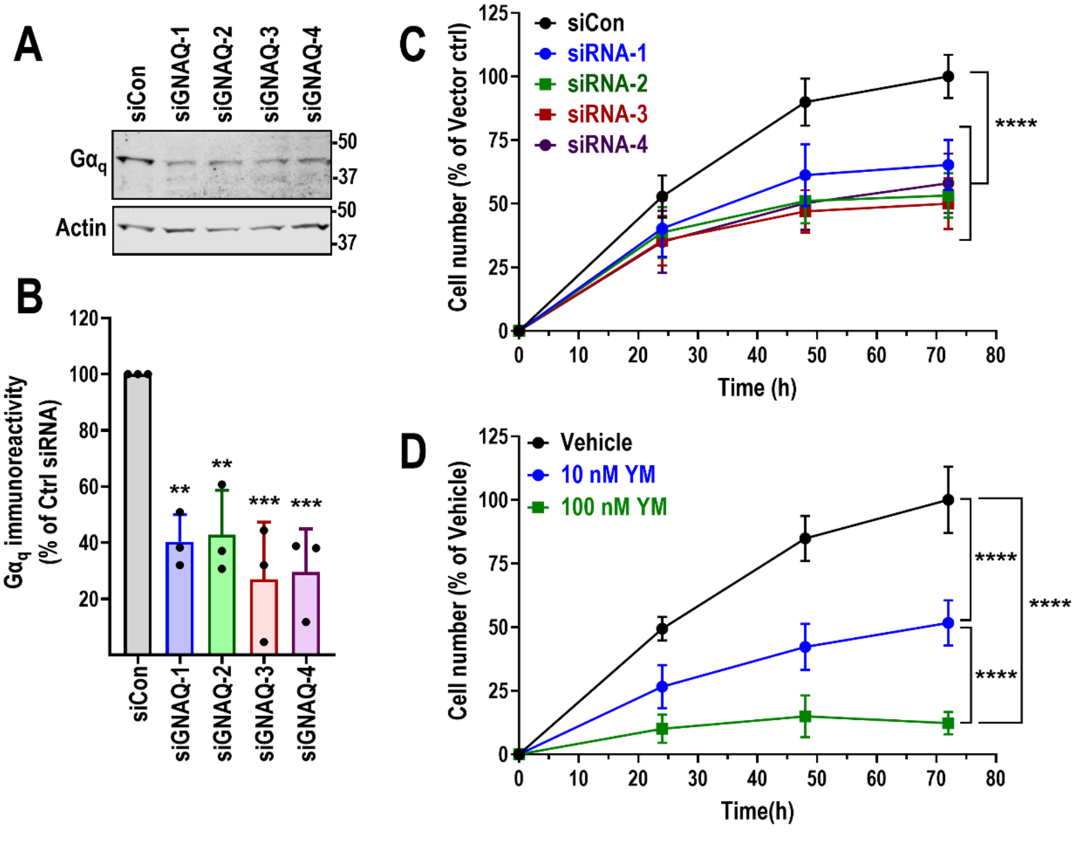

Figure S1. siRNA validation and YM-254890 concentration dependence. Representative western blot (**A**) and quantification of three independent experiments (**B**) demonstrating knock-down efficiency of GNAQ (encoding Gα$_q$) using the individual siRNA sequences in the SMART-POOL used in this study. $^{***}P<0.0001$ $^{****}P<0.0001$ using one-way ANOVA with Tukey's *post hoc* test for pairwise comparisons. **C.** All four individual siRNA result in significant decrease in 92.1 UM cell growth. Both the degree of knockdown and the magnitude of cell growth inhibition is lower than that achieved with the SMART-POOL. Result of three independent experiments with 5 technical replicates in each. **D.**

Treatment of 92.1 cells with 10 nM YM-254890 results in 50% decrease in cell growth. 100 nM results in almost complete inhibition of 92.1 cell growth. This is consistent with previously reported IC_{50} values. Result of two independent experiments with 5 technical replicates in each. $^{****}P<0.0001$ using two-way ANOVA with Tukey's *post hoc* test for pairwise comparisons.

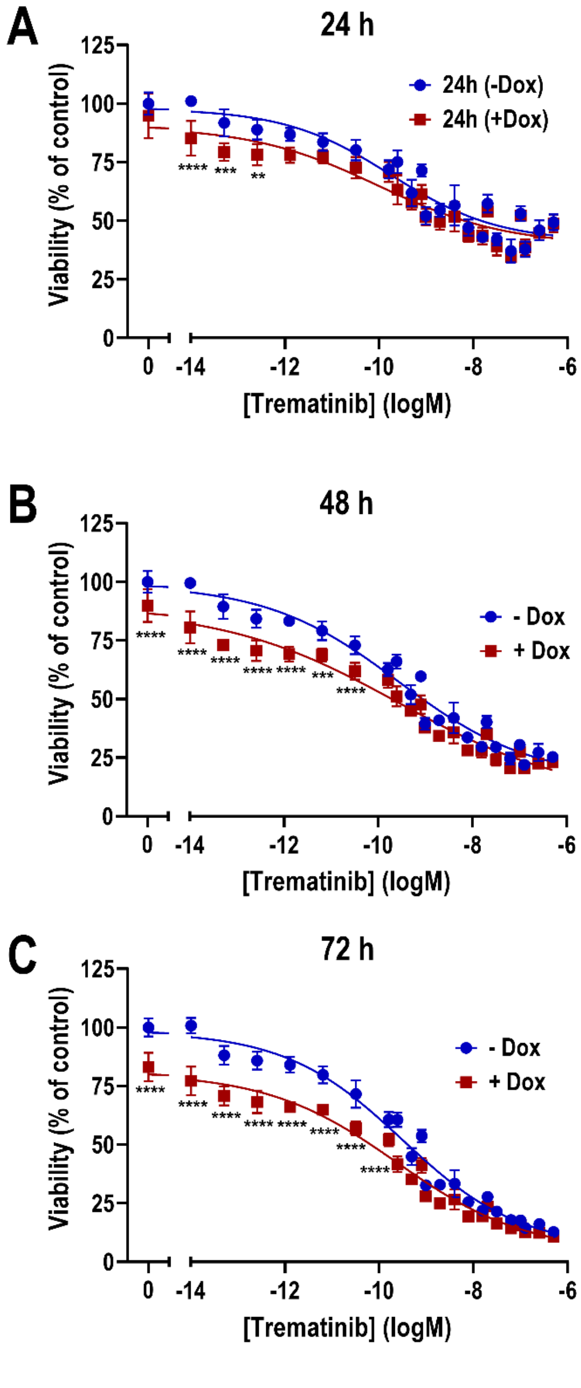

Figure S2. Dose-dependent effect of Trametimib (Tram) on 92.1 cell growth. Real-time Glo cell viability assays were performed in cells treated with increasing concentrations of Tram, with or without concurrent induction of RGS2 protein expression using Doxycycline (Dox; 1µg/ml) for 24 h (**A**), 48 h (**B**) and 72 h (**C**). At low concentrations of Tram, induction of RGS2 significantly enhances the suppression of cell growth. This effect is lost at concentrations >1 nM. The effect of RGS2 was more pronounced at

later time points. IC_{50} for Tram induced reduction in cell viability was not significantly changed by RGS2 protein expression induction. Results from three independent experiments run with triplicate samples. $^{**}P<0.01$, $^{***}P<0.001$, $^{****}P<0.0001$ using two-way ANOVA with Tukey's *post hoc* test for pairwise comparisons.
